# Supplementary material for: Reducing intraventricular hemorrhage following the implementation of a prevention bundle for neonatal hypothermia
Source: PLoS One. 2022 Sep 2;17(9):e0273946. doi: 10.1371/journal.pone.0273946 (PMC9439247; doi:10.1371/journal.pone.0273946)
Supplement: Attachment — Submitted filename: Response to Reviewers.docx [file pone.0273946.s003.docx]

Dear Editor:

We are submitting a revised version of the manuscript “*Reducing intraventricular hemorrhage following the implementation of a prevention bundle for neonatal hypothermia*” to be considered for publication in *PLOS ONE*.

In this revised version, we carefully addressed all the concerns raised by the editor and the reviewers, and we have provided a detailed response to all the comments below. The revised portions in the resubmitted manuscript have been denoted in red font.

Sincerely,

Po-Nien Tsao, MD, PhD

Professor, Department of Pediatrics

Director, Division of Neonatology

National Taiwan University Hospital

Vice Chairman, Research Center for Developmental Biology & Regenerative Medicine

National Taiwan University

No. 7, Chung-Shan South Road, Taipei 100, Taiwan

Tel: 886-2-23123456 ext 71013

Fax: 886-2-23147450

Email: tsaopn@ntu.edu.tw

**Response to editor**

1. Please ensure that your manuscript meets PLOS ONE's style requirements, including those for file naming. The PLOS ONE style templates can be found at <https://journals.plos.org/plosone/s/file?id=wjVg/PLOSOne_formatting_sample_main_body.pdf>, and <https://journals.plos.org/plosone/s/file?id=ba62/PLOSOne_formatting_sample_title_authors_affiliations.pdf>

**Response: We thank the reviewer for this important reminder. We have reviewed our manuscript thoroughly and ensured that the formatting is in order.**

2. In your Data Availability statement, you have not specified where the minimal data set underlying the results described in your manuscript can be found. PLOS defines a study's minimal data set as the underlying data used to reach the conclusions drawn in the manuscript and any additional data required to replicate the reported study findings in their entirety. All PLOS journals require that the minimal data set be made fully available. For more information about our data policy, please see <http://journals.plos.org/plosone/s/data-availability>.

**Response: We thank this timely reminder. We have uploaded our study’s data set under the Supporting Information files section.**

**Response to reviewers**

Reviewer #1:

- Title

The title is not parallel to the objectives of the study.

**Response: We thank the reviewer for this pertinent comment. We have revised our title to better reflect our study objectives, as suggested by the reviewer. Our new title is: “Reducing intraventricular hemorrhage following the implementation of a prevention bundle for neonatal hypothermia” and this has been included in our revised manuscript.**

- Abstract
  - Introduction: please describe about justification of the study?

**Response: We thank the reviewer for this timely reminder. We have revised our Introduction section to reflect the justification and objectives of the study, as suggested by the reviewer. Please refer to page 3, lines 31-32 in the manuscript (mark in red). We have established that hypothermia immediately after birth is a common, but difficult to prevent challenge among very low birth weight (VLBW) infants worldwide. For this reason, we have designed a protocol that acts as a prevention bundle to improve infant outcomes.**

- Methods: the study design and setting, sample size, data collection method, source population, eligibility criteria, data analysis and Etc. are not clearly described in the method section of the abstract.

**Response: We thank the reviewer for this pertinent comment. We have revised our Abstract session, as suggested by the reviewer. Please refer to page 3, lines 36-45. We have rewritten this part of our Abstract to ensure comprehension and readability.**

- Discussion/conclusion: please remove the word discussion. And what is the recommendation based on the findings of the study?

**Response: We thank the reviewer for this important comment. We have revised the section header, as suggested by the reviewer. Please refer to page 4, lines 54-57. We believe that this protocol is an effective, simple, low-cost, and replicable tool for most of the hospitals. Readers may refer to our algorithm to improve the outcomes of preterm hypothermia in their hospital.**

- Introduction

Define hypothermia here. Please show the gap in the introduction section of the manuscript.

**Response: We thank the reviewer for this comment. We have revised our Introduction section to highlight the definition of hypothermia, as well as provide more background and key literature related to our study. Please refer to page 4, lines 66-67 and page 6 lines 95-106. The World Health Organization (WHO) defines neonatal hypothermia as a temperature of less than 36.5 °C. We have also highlighted the high incidence of preterm hypothermia in developed countries.**

- Methods and materials

What is the word “general”? Please re-write the methods and materials section separately by incorporating the sub-element study period, study design, eligibility criteria, data collection procedure, sample size and sampling procedure, operational definition ….

Please operationalized your outcome variables and prevention bundle?

What does it mean the word feasible and effective? Please also operationalized this word?

In general, please re-write it clearly?

**Response: We thank the reviewer for these pertinent comments. We have revised our manuscript to reflect these improvements, as suggested by the reviewer. Please refer to page 7, lines 115-135. We have rewritten the study design and attached the algorithm for our prevention bundle (Fig 1) to ensure reader comprehension and readability.**

- Results

Why the pre-intervention and post-intervention groups are different in number?

**Response: We thank the reviewer for this question. This study was retrospective in nature and based on a previous project of quality improvement following national policy. The beginning and the ending of enrollment were both based on national policy, so the numbers of participants in each group (grouped based on their recruitment periods), could not be controlled. Additionally, the effect of prevention bundle in improving outcomes might fade gradually, because there would not be repeated intensive training for medical staff once the standardized SOP had been established, and their skill to execute the prevention bundle correctly as time goes on might be a reasonable concern. Thus, we tried to collect as many infants for the post-intervention group as we could, to highlight the effectiveness and sustainability of our prevention bundle**.

- Discussion

Please re-write based on the finding of the study

**Response: We thank the reviewer for this suggestion. We have revised our Discussion section to reflect the findings of our study, as suggested by the reviewer. Please refer to page 14 lines 221-227, 233-236, and 240-253 in the manuscript (mark in red). We also compared our study to a recent meta-analysis, cited more references and emphasized the clinical significance of reducing the IVH rate among infants after using our prevention bundle.**
