# Supplementary material for: Reducing intraventricular hemorrhage following the implementation of a prevention bundle for neonatal hypothermia
Source: PLoS One. 2022 Sep 2;17(9):e0273946. doi: 10.1371/journal.pone.0273946 (PMC9439247; doi:10.1371/journal.pone.0273946)
Supplement: Attachment — Submitted filename: New Microsoft Word Document (2).docx [file pone.0273946.s004.docx]

**Abstract**

**General comment: please correct the major editorial problem like alignment……..**

Introduction: In line 32 of the abstract section, there is a word…..difficult to prevent…... However, your study intended to investigate about the efficacy of the prevention bundles. The two things are contradicting each other’s.

Methods: what type of retrospective study does your study used? Please say something about sample size calculations in the methodology section of the manuscript. What are the study units? Is it VLBW, preterm neonates or both?

Result: Why the study intended to investigate only the stated short-term outcomes of hypothermia? Why other outcomes of hypothermia are not investigated yet?

**Methods**

Study design: please write introductory sentence about prevention bundle? When you say the prevention protocols are reliable and sound? How you are going to make the protocol standardized?

Outcomes: how you are going to measure hypotension and IVH?

Please re-write the methodology by considering what things has to be incorporated in each component of methods and materials? Why you classified the study group in to three? Why do not you classified as pre and post intervention group? Why the study groups differ in number?

**Results**

Low DR temperature: what is the parameter to increase the DR temperature from 19 to 21? Sentence in page 9, line 170-174 is not clear for reader, please write clearly? Institutional video: how do you assure the validity and reliability of the video? “….whereas the incidence of hyperthermia did not increase significantly between the two groups.” Delete this phrase?

**Discussion and conclusion**

WHO recommendation of DR temperature is 25 to 28. However, you upgrade DR temperature in your study setting from 19 to 21. Why?

The association of prevention bundle and mortality are not investigated yet in the study and you concluded that the prevention bundles are effective to reduce preterm mortality. However, the conclusions and interpretation has to be based on the finding of the study.
