# Supplementary material for: Reducing intraventricular hemorrhage following the implementation of a prevention bundle for neonatal hypothermia
Source: PLoS One. 2022 Sep 2;17(9):e0273946. doi: 10.1371/journal.pone.0273946 (PMC9439247; doi:10.1371/journal.pone.0273946)
Supplement: Attachment — Submitted filename: Response to Reviewers.docx [file pone.0273946.s005.docx]

5^th^ August 2022

Linglin Xie

Academic Editor

*PLOS ONE*

Dear Editor:

We are submitting a revised version of the manuscript “*Reducing intraventricular hemorrhage following the implementation of a prevention bundle for neonatal hypothermia*” to be considered for publication in *PLOS ONE*.

In this revised version, we have carefully addressed all the concerns raised by the editor and the reviewers, and we have provided a detailed response to all the comments below. The revised portions in the resubmitted manuscript have been denoted in red font.

Sincerely,

Po-Nien Tsao, MD, PhD

Professor, Department of Pediatrics

Director, Division of Neonatology

National Taiwan University Hospital

Vice Chairman, Research Center for Developmental Biology & Regenerative Medicine

National Taiwan University

No. 7, Chung-Shan South Road, Taipei 100, Taiwan

Tel: 886-2-23123456 ext 71013

Fax: 886-2-23147450

Email: [tsaopn@ntu.edu.tw](mailto:tsaopn@ntu.edu.tw)

**Response to editor**

1. **Please review your reference list to ensure that it is complete and correct. If you have cited papers that have been retracted, please include the rationale for doing so in the manuscript text, or remove these references and replace them with relevant current references. Any changes to the reference list should be mentioned in the rebuttal letter that accompanies your revised manuscript. If you need to cite a retracted article, indicate the article’s retracted status in the References list and also include a citation and full reference for the retraction notice.**

Response: We have reviewed and revised our reference list. (lines 333-345)

**Response to reviewer**

1. **General comment: please correct the major editorial problem like alignment……..**

Response: The format of the manuscript has been reviewed and revised by the authors and English-editoring company. (editing certificate is uploaded as supporting information)

2. **Introduction: In line 32 of the abstract section, there is a word…..difficult to prevent…... However, your study intended to investigate about the efficacy of the prevention bundles. The two things are contradicting each other’s.**

Response: We have substituted the phrase “difficult to prevent” with “common even in countries rich in medical resources.” (line 32)

3. **Methods: what type of retrospective study does your study used? Please say something about sample size calculations in the methodology section of the manuscript. What are the study units? Is it VLBW, preterm neonates or both?**

Response:

(1) We have revised the Methods section of the manuscript. The study was a quality-improvement project. (line 36)

(2) We anticipated enrolling 100 cases to achieve a 40% reduction in the admission hypothermia rate while considering a previous study, the duration of this QI project provided by the government, and the average number of VLBW infant births per month in our hospital. (lines 114-117)

(3) We recruited every newborn with a BBW <1,500 gm or GA <31 weeks during the study period. (lines 113-114)

4. **Result: Why the study intended to investigate only the stated short-term outcomes of hypothermia? Why other outcomes of hypothermia are not investigated yet?**

Response: Other short-term complications with relatively low prevalence, such as necrotizing enterocolitis or late-onset sepsis, were not investigated considering our study period and sample size. (lines 147–148)

5. **[Methods] Study design: please write introductory sentence about prevention bundle? When you say the prevention protocols are reliable and sound? How you are going to make the protocol standardized?**

Response:

(1) We aimed to design a standard hypothermia prevention bundle, which is a simple set of evidence-based practices that improve the reliability of their delivery and improve patient outcomes when implemented collectively, to prevent hypothermia after birth and investigate the bundle’s efficacy and short-term outcomes of VLBW infants. (lines 105-108)
(2) The final version of the prevention bundle, which serves as the intervention used in this study, was established and presented as an algorithm by the end of September 2017 to assure standardization. (lines 126-128)
6. **Outcomes: how you are going to measure hypotension and IVH?**

Response: IVH of any grade, revealed by routine cranial ultrasound, was included; hypotension was defined as mean blood pressure (mmHg) below the post-menstrual age (weeks) and requiring inotropic agents; acidosis was defined as an initial blood gas pH of <7.2. (lines 147–151)

7. **Please re-write the methodology by considering what things has to be incorporated in each component of methods and materials? Why you classified the study group in to three? Why do not you classified as pre and post intervention group? Why the study groups differ in number?**

Response: We have revised and rephrased the Methods section as suggested, including combining the pre- and intra-intervention groups into one group, the thinking process by which the algorithm was formed, and the reason for recruiting certain case numbers.

8. **Low DR temperature: what is the parameter to increase the DR temperature from 19 to 21? Sentence in page 9, line 170-174 is not clear for reader, please write clearly? Institutional video: how do you assure the validity and reliability of the video? “….whereas the incidence of hyperthermia did not increase significantly between the two groups.” Delete this phrase?**

Response:

(1) The higher the DR temperature, the easier it was to warm preterm babies. However, the following factors were also considered while regulating the DR temperature: the comfort of the obstetrician and the challenge of avoiding sweating to prevent the spread of infection during delivery. Thus, a higher DR temperature might not always be better, especially if there are other means to keep the baby warm. After negotiating with the obstetricians, we decided to increase the DR temperature from 19°C to 21°C as soon as we were notified about a pending preterm delivery. (lines 165–171)
(2) Line 180-185 is caption and legend of Figure 3.
(3) We used a checklist to assure the validity and reliability of the video. The completion rate was above 90%. (lines 191-192)
(4) We have deleted the phrase, as suggested.
9. **WHO recommendation of DR temperature is 25 to 28. However, you upgrade DR temperature in your study setting from 19 to 21. Why?**

Response: The higher the DR temperature, the easier it was to warm preterm babies. However, the following factors were also considered while regulating the DR temperature: the comfort of the obstetrician and the challenge of avoiding sweating to prevent the spread of infection during delivery. Thus, a higher DR temperature might not always be better, especially if there are other means to keep the baby warm. After negotiating with the obstetricians, we decided to increase the DR temperature from 19°C to 21°C as soon as we were notified about a pending preterm delivery. (lines 165–171)

10. **The association of prevention bundle and mortality are not investigated yet in the study and you concluded that the prevention bundles are effective to reduce preterm mortality. However, the conclusions and interpretation has to be based on the finding of the study.**

Response: We have revised our wording as suggested. We believe that this low-cost prevention bundle is a simple, replicable, and robust tool for decreasing the incidence of hypothermia and associated complications among VLBW infants in hospitals around the world. (lines 259–261)
